# Differentiation of Apple Varieties and Investigation of Organic Status Using Portable Visible Range Reflectance Spectroscopy

**DOI:** 10.3390/s18061708

**Published:** 2018-05-25

**Authors:** Jordan Vincent, Hui Wang, Omar Nibouche, Paul Maguire

**Affiliations:** 1 School of Computing and Mathematics, University of Ulster, Shore Rd, Newtownabbey BT37 0QB, UK; h.wang@ulster.ac.uk (H.W.); o.nibouche@ulster.ac.uk (O.N.); 2 School of Engineering, University of Ulster, Shore Rd, Newtownabbey BT37 0QB, UK; pd.maguire@ulster.ac.uk

**Keywords:** spectroscopy, pattern recognition, PLS-DA, apple, food authentication

## Abstract

Food fraud, the sale of goods that have in some way been mislabelled or tampered with, is an increasing concern, with a number of high profile documented incidents in recent years. These recent incidents and their scope show that there are gaps in the food chain where food authentication methods are not applied or otherwise not sufficient and more accessible detection methods would be beneficial. This paper investigates the utility of affordable and portable visible range spectroscopy hardware with partial least squares discriminant analysis (PLS-DA) when applied to the differentiation of apple types and organic status. This method has the advantage that it is accessible throughout the supply chain, including at the consumer level. Scans were acquired of 132 apples of three types, half of which are organic and the remaining non-organic. The scans were preprocessed with zero correction, normalisation and smoothing. Two tests were used to determine accuracy, the first using 10-fold cross-validation and the second using a test set collected in different ambient conditions. Overall, the system achieved an accuracy of 94% when predicting the type of apple and 66% when predicting the organic status. Additionally, the resulting models were analysed to find the regions of the spectrum that had the most significance. Then, the accuracy when using three-channel information (RGB) is presented and shows the improvement provided by spectroscopic data.

## 1. Introduction

Food fraud, the practice of adulterating or mislabelling food, is a global problem. This is illustrated by news reports detailing incidents from China [1], America [2] and the European Union (EU) [3]. In 2013, 4.6% of beef product samples tested by EU member states found that horse meat had been included undeclared [4]. In a subsequent test of honey, the EU found 14% of submitted samples to be ‘suspicious of being non-compliant’ [5]. All of these represent significant occurrences of food fraud, the economic impact of which is difficult to quantify. The Food Standards Agency (FSA) attributed part of this difficulty to the lack of reporting, as those affected often never notice anything amiss [6]. In particular, these incidents underscore the need for thorough and increasingly accessible methods of authenticating that food is as declared. This study looks at the use of a portable visible range spectrometer (380–700 nm) for potential applications in widely-accessible food authentication.

### 1.1. Food Authentication

Food fraud covers a range of acts such as mislabelling, diluting liquids to increase the sellable quantity, adulterating food products with additional compounds that should not be present and even selling products that are unfit for consumption. In the U.K., the National Food Crime Unit defines food fraud as ‘a dishonest act or omission, relating to the production or supply of food, which is intended for personal gain or to cause loss to another party’ [7].

Food authentication is concerned with the methods and techniques for validating food is as claimed, and it can be used as an approach to detecting food fraud. As food fraud can encompass such a wide variety of issues, there is consequently a wide range of determinations that can be used in food authentication. These determinations range from the variety of food or its organic status, through to its geographical origin or component concentration.

Due to the wide variety of determinations that can be of interest, there is a variety of different technologies and methods used in food authentication [8]:

- Spectroscopy (vibrational and florescence)
- Genomics and proteomics
- Liquid/gas chromatography and mass spectrometry
- Isotopic ratios
- Chemometrics and bioinformatics

Each method has its advantages and disadvantages and is capable of making different determinations with differing accuracy. Most of these methods are well tested and have been used to explore many different food authentication tasks (see [9,10] for examples).

One impediment in food authentication is the lack of a global, or even widely-accepted definition of what constitutes food fraud. For example, even within the EU, which often attempts to harmonise these types of regulation, there is no defined shared definition of the term food fraud [11]. The lack of a unified definition can hinder research as there is no shared goal of determinations to work towards.

Additionally the hardware required by the majority of the methods and the complexity of the analysis pose barriers to adoption throughout the supply chain. The hardware is traditionally large and immobile, limiting it to dedicated labs. One exception to this is spectroscopy, where the hardware is becoming increasingly small and portable [12]. The portable technology however still leaves the task of analysing the resulting data. Traditionally, in situ and external laboratories wishing to use food authentication techniques have been required to train and employ specialised technicians to perform the analysis.

The primary aim of this work is to investigate the performance of a machine-learning/pattern recognition approach to the data produced by the more portable spectrometer technology to alleviate the barrier posed by the complex data analysis. The documented food fraud incidents demonstrate there is a use-case for more portable and accessible food authentication methods.

### 1.2. Spectroscopy

Spectroscopy can be split into different areas based on the area of the spectrum utilised such as ultra-violet, visible, near-infrared and mid-infrared. Significant work has taken place recently with near-infrared spectroscopy in the context of food authentication as the hardware is considered portable by industrial standards and can be easily taken into the field, such as [13,14]. Visible range spectroscopy also has been used in determining food quality attributes, such as ripeness [12], but there has been little work with the specific objective of food authentication.

### 1.3. Visible Range Spectroscopy

In addition to the primary aim of investigating the performance of a machine learning approach, this study also addresses this gap and begins to take a look at visible range spectroscopy for the purpose of food authentication. Visible range spectroscopy was chosen for the following reasons:

- The spectrometer is consumer portable, not just portable by industrial standards
- It is significantly less expensive than the other methods
- The testing procedure is non-destructive (i.e., it does not damage produce)

Portability and cost are important factors that have played a part in preventing the utilisation of food authentication techniques across the supply chain. More importantly, portability and size are key requirements for the system to be adopted by consumers. Devices that are difficult to move or not easily handled are unlikely to enter the consumer market on ergonomic grounds. A non-destructive test would be advantageous as all produce can be tested and then sold. This is an improvement over destructive methods where only a small sample of each batch can be tested.

## 2. Related Work

This section reviews some of the related work either with similar methods or with similar goals.

### 2.1. Portable Bluetooth Spectrometer

Das et al. [15] presents a portable spectrometer that communicates with a smartphone using Bluetooth. The spectrometer has a range of 340 nm–780 nm, putting it in the visible range. The authors validated their portable design by applying it to ripeness testing of fruit (apples) using the ultraviolet (UV) fluorescence signal from chlorophyll tracked over 12 days. They then performed a linear fit for the chlorophyll signal over time. Their device utilised a nozzle to shield the spectrometer from ambient light interference. Overall, they concluded that ‘a satisfactory agreement was observed between ripeness and fluorescence signals’. They also compared the spectra produced by their hand-held device illuminated by LEDs to the spectra produced by larger spectroscopy hardware; concluding there was a strong correlation between the produced spectra. However, rather than investigate the results in a setting with some ambient light interference as done in this research, they shielded the device from ambient light interference.

Additionally, the SCiO, a commercial venture by Consumer Physics [16], is a small portable Bluetooth spectrometer. Unlike Das et al., however, the spectrometer has a range of 740–1060 nm [17] which puts it in the near-infrared range instead of the visible range, which is the focus of this study.

### 2.2. Prediction of Apples Organic Status with NIRS

Song et al. [13] presented a work to determine if near-infrared spectroscopy could correctly determine an apple’s organic status. They looked at the reflection spectra for the apples in the near-infrared range. Each chosen point on an apple was scanned three times and averaged together to produce a sample. Pre-processing consisted of baseline correction and standard normal variate (SNV) normalisation with PLS-DA used to produce a model. For validation, 10-fold cross-validation was used. They found that the accuracy when determining organic status was greater than 95%. This study is an extension of that research, assessing a portable visible range spectrometer for similar purposes.

### 2.3. Visible Range Spectroscopy and Foodstuffs

In addition to the previous studies that focused on portability, there has been significant work on the utility of visible range spectroscopy to determine food quality attributes. A common theme is to combine visible range spectroscopy with near-infrared spectroscopy for the additional information it can provide. The combination of the two has been used to assess beer quality during the fermentation process [18]; carotenoid contents in banana fruit pulp [19]; and quality parameters of grapes [20]. Preprocessing methodologies in the studies included smoothing, baseline removal, first/second derivatives of the spectra and multiplicative scatter correction. Overall, the authors of these studies all concluded that their systems were successful at their task and that the predictions showed a good correlation with their target.

There are a significant number of components of many fruits and vegetables that have absorption or emission bands in the visible range of the spectrum [21]. This gave reason to believe that the visible range is worth investigating.

## 3. Experiment Design and Data Collection

**Figure 1:**
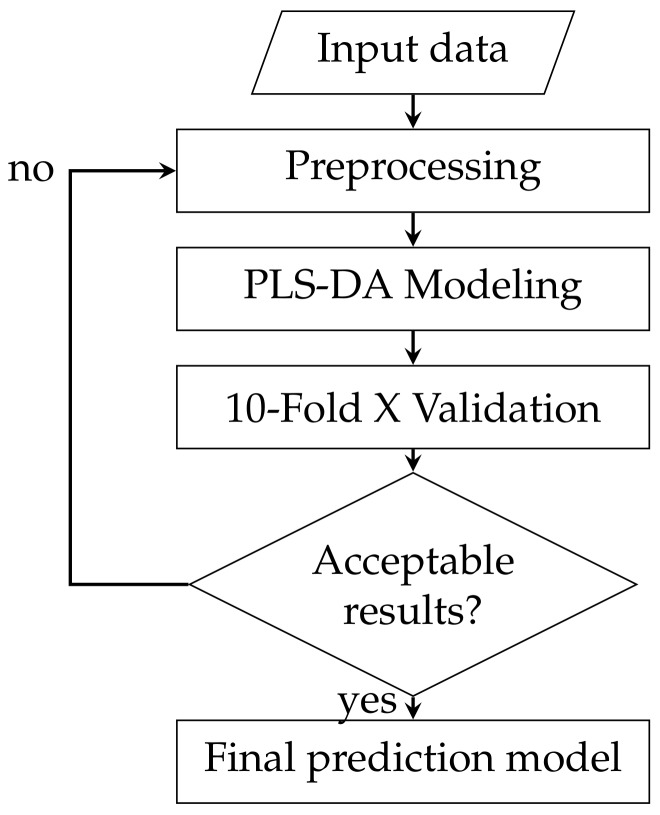
Pattern recognition process.

The pattern recognition process is iterative, with different preprocessing and models trialled to see which performs best with the data and its underlying features. For illustration purposes a flowchart of the high level processing pipeline is in Figure 1. Prepossessing attempts to ‘clean’ the data and remove features from the data that are known or thought to be irrelevant. The modelling stage uses the preprocessed data to build a model that can be used to predict outputs in the future. Finally the validation stage tests the model to assess its performance. If the performance is unsuitable, the process begins again with alterations to either the preprocessing or modelling stage, with the goal of either cleaning the data further or finding a model that better represents the data.

### 3.1. Spectrometer-OceanOptics SPARK

The spectrometer used in this paper is the OceanOptics SPARK. It is a visible range USB spectrometer with a wavelength range of 380–700 nm and a spectral resolution of 4.5–9.0 nm [22]. It was chosen as it is portable, has a wavelength range that covers the whole of the visible spectrum and has a price that makes it cost effective for the entire supply chain (including consumers) to afford.

### 3.2. Choice of Apples

To assess the performance of the machine learning approach, two determinations were chosen. The first is the type of apple, which requires assessing properties related to the apple. The second is organic status, a more nuanced determination. Such a determination will require identifying any indication (direct or indirect) that pesticides were used when growing the apple.

Multiple varieties of apples were chosen: Gala, Braeburn and Pink Lady. They were chosen as they give a range of prices that are distinctive enough that there is merit in telling them apart from each other, as well as their organic/non-organic status. For this study, 132 apples were used overall, with 44 apples of each type. Half of each type was organic and the other half non-organic. In addition, within each class, there were at least two countries of origin to provide geographic variety; the country breakdown can be seen in Table 1.

The prices for each can be seen in Table 2. In particular, there is utility in telling Pink Lady apart from Gala and Braeburn apples and telling all organic types from their non-organic types, due to the price differences. Pink Lady apples are 65% more expensive than the Gala and Braeburn apples, and the organic varieties are on average 43% more expensive than their non-organic counterparts.

### 3.3. Nature of the Data

The data produced by the spectrometer are collinear, i.e., the value of each attribute is not fully independent of those near it. This is primarily due to the sensor hardware and its spectral resolution.

In addition, one property of interest is the system’s sensitivity to ambient conditions, given consumers could not be expected to have as much control over the environment as a lab. To test this, only simple attempts were made to restrict the ambient conditions during scans. The lights were off and the blinds closed, but light from outside was still present. This is unlikely to cause any issues in isolation, as all scans from the same ‘session’ will have the same baseline interference. Therefore, to assess the sensitivity, the apples were re-scanned on a second day with different ambient conditions, and the model from the first session was tested with this second session. This ensures that the model is robust enough to overcome any noise.

### 3.4. Data Collection

Four samples were taken of different parts of each apple to account for any surface changes/defects and provide a better overall picture. Each sample comprised three scans, which were averaged together to reduce noise. As the SPARK sensor has no built-in light source, a tungsten-halogen lamp from a VIS-NIR probe was used to provide illumination as it emits across the visible range of the spectrum [23]. Each sample corresponds to a row in the input matrix, and each column corresponds to a sample’s wavelength.

### 3.5. Data Preprocessing

Before being passed into the classification model, the raw spectral data were preprocessed. In keeping with the portable theme, the preprocessing methods were kept to those that had the lowest computational complexity possible.

The first step was to subtract the minimum value of each data point (attribute) from the others in the sample. This ensures the minimum value in each spectra is zero and accounts for any changes in the minimum light intensity over the entire range. In addition to being a very simple operation with low computational complexity, it also has the advantage that it does not require parameter optimisation as some of the more complex methods of baseline removal do.

The second preprocessing step was to normalize the data. This was done as the absolute attribute value is not of interest. It primarily varies with distance between the sensor, light source and object. It is also not expected to be consistent over multiple days due to changes in the ambient conditions. Again, with an eye towards computational complexity, a two-norm (vector normalisation/Euclidean norm) was used, which normalises the data such that all columns sum to one:

(1) $$x_{i}=\frac{x_{i}}{\sqrt{\sum_{i=1}^{n}x_{i}^{2}}}.$$

Finally, the data were smoothed with a Savitzky–Golay filter using a polynomial order of three and a window size of 31.

**Figure 2:**
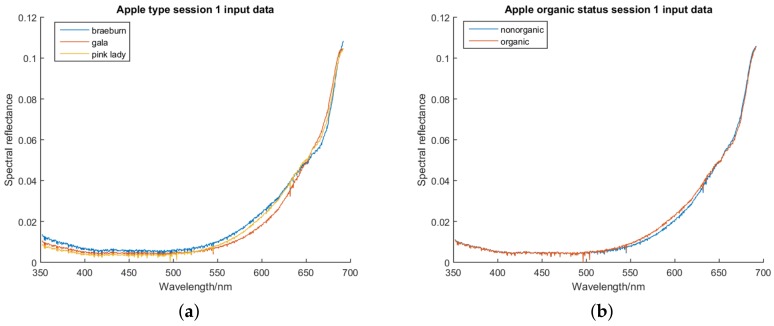
(**a**) Graph of the average apple spectra for each apple type after preprocessing. (**b**) Graph of the average apple spectra for each organic/non-organic class after preprocessing.

Graphs of the average preprocessed data for the first session, coloured based on class, can be found in Figure 2.

### 3.6. Data Analysis

Partial least squares discriminant analysis (PLS-DA) was used as the primary analysis/classification algorithm. It was chosen because it is a common and well-tested algorithm in the chemometrics field for dealing with spectral data.

There are different implementations of PLS-DA, and the one used in this paper is SIMPLS [24]. It is worth noting that this implementation centres the columns (wavelengths/variables) of the input matrices before running. The SIMPLS implementation in MATLAB (plsregress) was used.

The training process for PLS-DA involves finding a linear transformation of the input matrix that maximises its covariance with the output matrix. PLS-DA has one parameter, which is the number of components it is allowed to create from the input data. There is no catch-all answer to the best number of components, and more is not always better, as it may over-fit the data. Thus, it is necessary to determine the optimum number for the task at hand through appropriate trials and verification.

In this study, two tests are used with each dataset. The first is the ‘single session’ test, which cross-validates data obtained from a single recording session. The second test is the ‘inter-session’ test, which uses data recorded in a different recording session, with differing ambient light conditions, as a test set and the first sessions data as a training set.

Together, these provide an assessment of the system’s sensitivity to changes in the ambient conditions and its suitability for use outside of a highly controlled lab environment. Performance of the system is measured relative to a ‘ZeroR’ baseline, the accuracy that would be achieved if the system consistently guessed the majority class in the dataset.

## 4. Single Session Experiment

In this experiment, 10-fold cross-validation was used to get the accuracy for each model tested. The cross-validation was itself repeated 10 times with different random partitions (producing 100 results total) in order to get the most accurate profile of performance possible. Models were tested with a number of PLS latent variables from one to fifteen.

### 4.1. Type Differentiation

**Figure 3:**
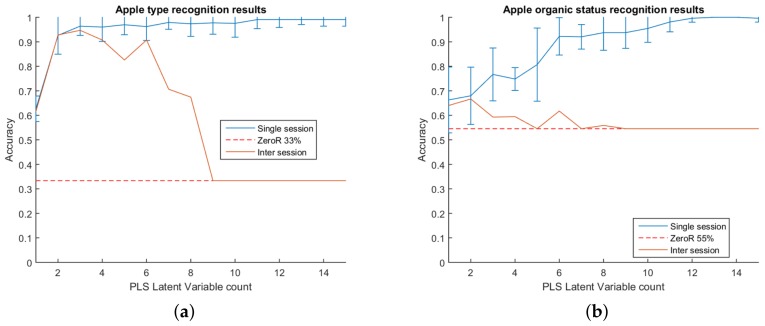
Graph of the accuracy for (**a**) predicting the apple type and (**b**) predicting the apple organic status. ZeroRrepresents the accuracy achieved if the predictor were to consistently guess the majority class and is used as a baseline.

The first test was for the system to differentiate between the type of apple scanned. When differentiating between the type of apples, the accuracy quickly rises to above 90% at two PLS components and continues to rise, although more slowly, as more components are added. The system achieves 96% accuracy at three components and continues to slowly climb. The results for the experiment are shown in Figure 3. The point represents the mean accuracy of the 100 tests, and the error bars extend two standard deviations from the mean. Overall, the system was very successful at predicting the apple type in this test.

### 4.2. Organic / Non-Organic Differentiation

The second test of the system was to differentiate between the organic status of the scanned apples. An organic apple will be physically indistinguishable from a non-organic apple; therefore, any differences picked up are expected to originate either directly or indirectly from the effects of the pesticides. Compared to the previous test of differentiating types, the system was less effective. Accuracy was slower to rise as components were added than it was for apple types, achieving 90% accuracy at seven components. The results for the experiment are shown in Figure 3.

## 5. Inter-Session Experiment

The next experiment is the inter-session tests. As mentioned previously for this experiment, the apples were re-scanned following the same procedure on a different day. The data used in the single session test were used as training data for the model, and the newly-scanned data were used as a test set. The purpose of this test is to gauge how sensitive the system is to changes in the ambient conditions of the scans and to verify that PLS-DA has identified accurate and relevant signals rather than just picking up on well-correlated background noise.

### 5.1. Type Differentiation

The inter-session results for differentiating type track well with the single session results and are shown in Figure 3. The accuracy rises along with the single session results up to three components, staying within the error bars of the single session test. After three PLS components, the accuracy decreases slightly, then after six, declines rapidly. Overall however, the results are encouraging, with a peak accuracy of 94% at three components representing a 61 percentage point increase from the ZeroR baseline.

### 5.2. Organic / Non-Organic Differentiation

The results for the inter-session organic/non-organic differentiation did not track well with their respective single session results. The best accuracy achieved was 66% at two components, after which the inter-session performance fell before following the baseline. Overall, the best accuracy represents an improvement of 12 percentage points from the baseline.

## 6. Model Analysis

This section takes a more detailed look at the PLS model that was produced and on which areas of the input data it put the most weight.

### 6.1. VIP Score

The metric used to quantify the importance of each wavelength in the model is the variable importance in projection (VIP) score. In PLS, each wavelength (or attribute) has a weight for each component, and each PLS component has a metric for the amount of variance it explained. The VIP score is a weighted sum of each wavelength’s weight over all components, weighted by the variance explained in each component [25].

#### 6.1.1. Apple Type Models

**Figure 4:**
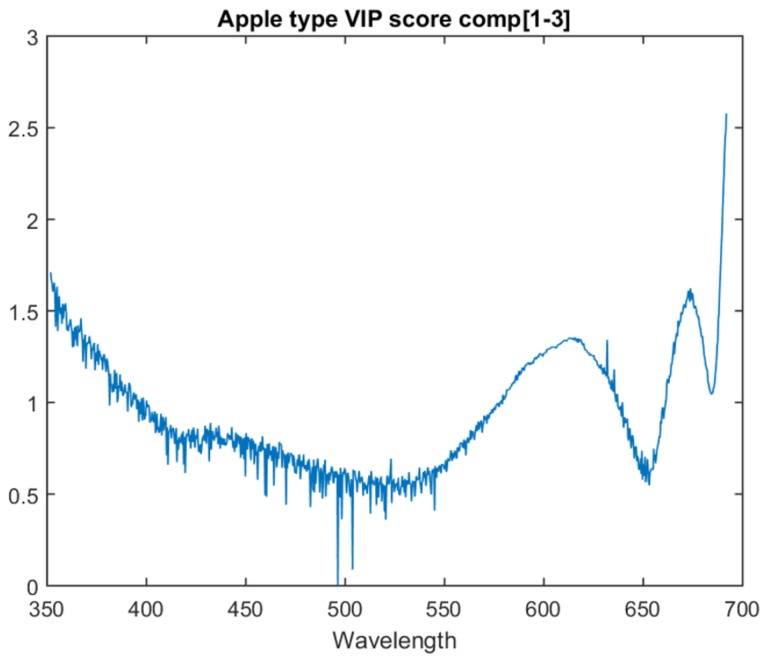
VIP score for the first three components of the apple type model.

Of the various apple type models, the one with the highest inter-session accuracy is at three PLS components. The graph for the VIP score of each attribute for this model is in Figure 4. A higher value indicates a higher importance. There are three main distinctive features. The first is that the highest weighted attribute is the one closest to the near-infrared region, at the top limit of what the spectrometer is capable of seeing (691 nm). The second is a peak in importance shortly before this at approximately 673 nm. The third is the smaller, but still noticeable peak at approximately 615 nm.

A detailed breakdown of these features is left for future work. However, it is noted that chlorophyll A can be observed around 662 nm, i.e., within the bounds of the second feature. It is also noted that the area around 500 nm shows a number of significant instantaneous changes in score. Due to the co-linearity of the data, adjacent attributes are not expected to exhibit such instantaneous differences in score.

#### 6.1.2. Apple Organic Status Models

**Figure 5:**
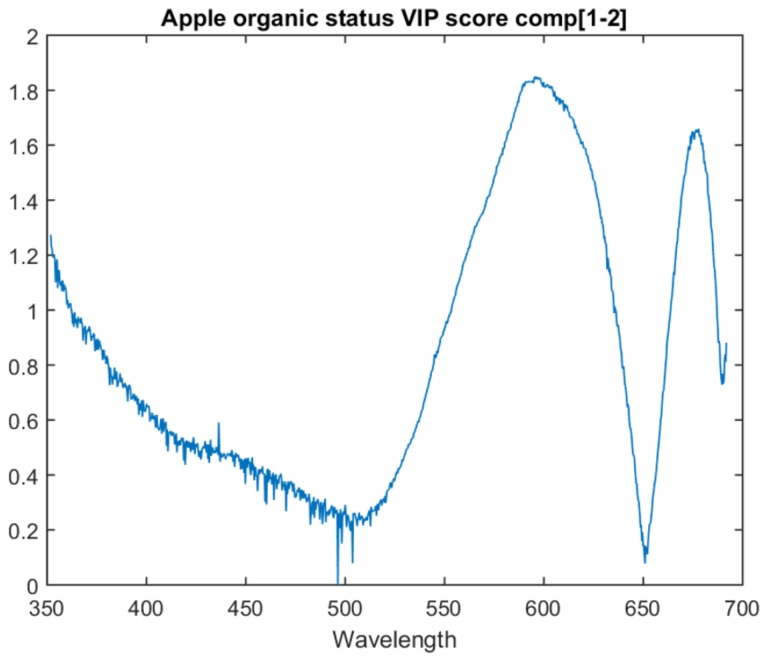
VIP score for the first two components of the apple organic status model.

The organic/non-organic model VIP scores are focused on the two PLS latent variable model as it achieves the best inter-session accuracy. The graph for the VIP score of each attribute for this model is in Figure 5.

This model follows a similar trend to the type model. It has two primary peaks at approximately 673 nm and another before it at approximately 597 nm. When compared with the apple type model, the end of the spectra is not weighted as significantly relative to the two main peaks. This model also contains spikes/drops one attribute in width, similar to the type model around 500 nm.

## 7. Comparison of Spectral Information vs. RGB Colour Information

Three-colour channel information such as RGB would not be sufficient to achieve our results when predicting apple type. Had a picture of the apple been taken at these points and the pixel RGB values used, the accuracy of such a system would be significantly lessened. To evidence that colour information from a camera would not be sufficient to achieve our results, we have converted the spectral samples into RGB using the profile of various commercial camera models.

All cameras, screens or other recording/display devices have a profile of sensitivity vs. wavelength for each colour channel. The channel value is the weighted sum of the different wavelengths, each wavelength weighted by its sensitivity. With this profile and the spectra, we can convert the wavelengths into the RGB channels as various devices would see/display them. To perform this test, the spectra were converted into RGB using the sensitivities database provided by [26].

Converting the spectra into RGB using these camera profiles and then running the training and testing produces the results shown in Table 3. The best result using the three RGB channels instead of the raw spectra was 63%, achieved with the profile of a Point Grey Grasshopper 50S5C.

## 8. Other Models (One vs. All)

In addition to the previous models, which attempted to be multi-purpose, there were additional two-class (one vs. all) models trialled. Three models were produced, one for each apple type, which aimed to only predict whether an apple was that type or not (one vs. all). As the apple type predictions proved successful, predicting apple types was the focus of these additional models.

### 8.1. Accuracy

The one vs. all models produce varying improvements over the multiclass model. All of the one vs. all models were capable of achieving an accuracy greater than or equal to 90% in both the single session test and inter-session test. The optimum number of PLS latent variables varied.

As the classes are now unbalanced, the baseline accuracy for each apple type class is 33%, and the baseline accuracy for each non-type class is 66%. The optimum inter-session accuracies for the models were 94%, 96% and 93% for the Gala, Braeburn and Pink Lady models, respectively. All three models showed some pattern of deterioration in the accuracy should PLS be allowed to have many latent variables. This deterioration was not present in the single session results.

As the classes are now unbalanced, it is also beneficial to take a look at the confusion matrices, which can be seen in Table 4. Both the Gala and Braeburn confusion matrices show the same trait of a 0% false positive rate with respect to identifying apples that were not the target for prediction.

### 8.2. Model Analysis

The one vs. all models show similar traits in variable importance to the multiclass model. All three one vs. all models have the last attribute as the most important and have a similar peak just before it. This suggests that there is relevant information in these two areas for all three determinations.

**Figure 6:**
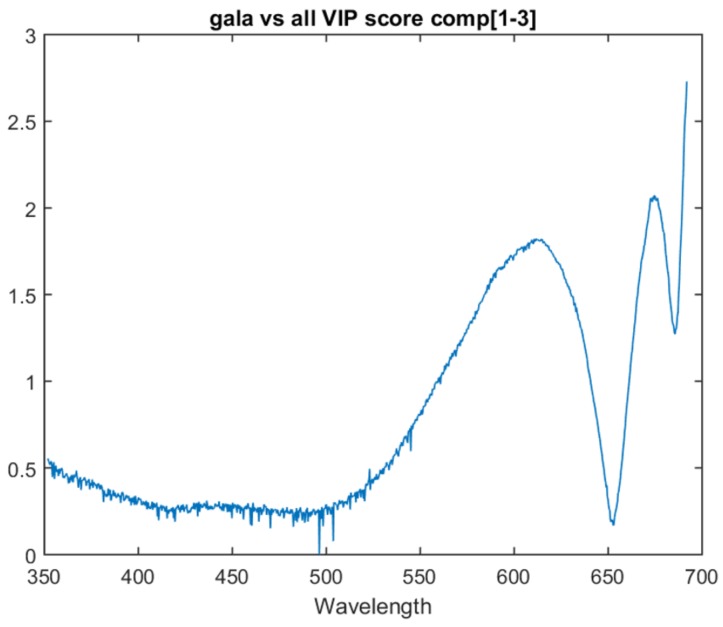
VIP score for the the Gala vs. all model at three components.

**Figure 7:**
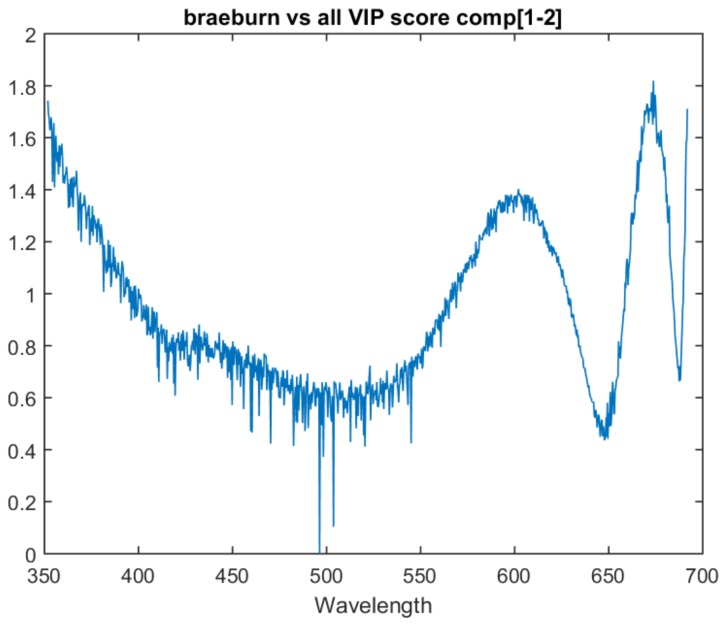
VIP score for the the Braeburn vs. all model at two components.

**Figure 8:**
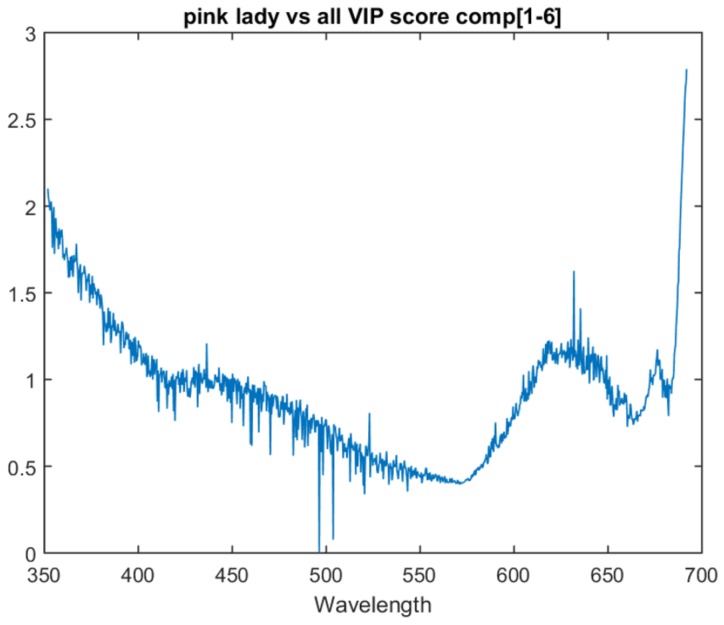
VIP score for the the Pink Lady vs. all model at six components.

The Pink Lady vs. all model lacks the peak that was present at 675 nm in the previous models. Instead, it has a significant upward trend in variable importance as it approaches the bottom area of the spectrum. This upward trend is also present in the Braeburn vs. all model. The one vs. all model VIP scores for Gala, Braeburn and Pink Lady can be found in Figure 6, Figure 7 and Figure 8 respectively. All three models’ VIP scores were taken at the number of PLS components where the inter-session accuracy was highest.

## 9. Conclusions

This paper investigated the combination of low cost visible range spectrometers (380–700 nm) and pattern recognition applied to the task of differentiating apple types and their organic status. With minimal preprocessing and using partial least squares discriminant analysis for classification, the system was successfully able to differentiate the three types of apples Gala, Braeburn and Pink Lady. It achieved over 93% accuracy even in the presence of mild ambient interference. Further work could investigate alternate preprocessing and classification algorithms to ascertain to what extent the performance of this method can be improved.

The organic/non-organic tests were less successful and overall, the system struggled to make meaningful determinations about the apples’ organic status. Further work is required to ascertain if this is due to a lack of relevant information in the visible range of the spectrum or because more complex preprocessing is required.

Additionally, this paper explored some of the main features found in the produced PLS models and highlights regions of the spectrum that appear to be the most significant. The model examination suggests the most important attributes are three regions at approximately 961 nm, 673 nm and 615 nm when determining apple types. Future work could investigate these areas of the spectrum in order to ascertain what specifically is present in these areas.

Overall, the results demonstrate that the VIS spectroscopy-based system has merit and warrants further investigation to identify its limits.

## Figures and Tables

**Table 1:** Breakdown of the countries of origin when differentiating (a) apple type and (b) organic status.

(a)				(b)	
Country	Gala	Braeburn	Pink Lady	Organic	Non-Organic
Austria	20			20	
France		24	24		48
Germany		20		20	
Italy			20	20	
U.K.	24				24

**Table 2:** Prices per kg for each apple type.

Type	Non-Organic	Organic
Gala	£2.20/kg	£3.18/kg
Braeburn	£2.20/kg	£3.18/kg
Pink Lady	£3.65/kg	£5.12/kg

**Table 3:** Results of classification with RGB based on camera spectral sensitivity functions.

Camera	Accuracy
Canon 1D MarkIII	60%
Canon 5D MarkII	62%
Canon 50D	61%
Canon 500D	62%
Nikon D3	59%
Nikon D90	59%
Nokia N900	58%
Nikon D5100	60%
Point Grey Grasshopper 50S5C	63%

**Table 4:** Confusion matrices for one vs. all models taken at their highest accuracy. Three, two and six components for Gala, Braeburn and Pink Lady, respectively.

Gala	Not Gala	Braeburn	Not Braeburn	Pink Lady	Not Pink Lady
84.7%	15.3%	89.8%	10.2%	97.2%	2.8%
0%	100%	0%	100%	2.6%	97.4%

## Abbreviations

The following abbreviations are used in this manuscript:

PLS-DA	Partial last squares discriminant analysis
VIP	Variable importance in projection
VIS	Visible electromagnetic range
NIR	Near-infrared electromagnetic range
